# Degradation of Acephate and Its Intermediate Methamidophos: Mechanisms and Biochemical Pathways

**DOI:** 10.3389/fmicb.2020.02045

**Published:** 2020-08-18

**Authors:** Ziqiu Lin, Shimei Pang, Wenping Zhang, Sandhya Mishra, Pankaj Bhatt, Shaohua Chen

**Affiliations:** ^1^State Key Laboratory for Conservation and Utilization of Subtropical Agro-bioresources, Integrative Microbiology Research Centre, South China Agricultural University, Guangzhou, China; ^2^Guangdong Laboratory for Lingnan Modern Agriculture, Guangzhou, China

**Keywords:** toxicology, physicochemical degradation, microbial degradation, degradation pathways, degradation mechanisms, gene

## Abstract

Acephate is an organophosphate pesticide that has been widely used to control insect pests in agricultural fields for decades. However, its use has been partially restricted in many countries due to its toxic intermediate product methamidophos. Long term exposure to acephate and methamidophos in non-target organisms results in severe poisonous effects, which has raised public concern and demand for the removal of these pollutants from the environment. In this paper, the toxicological effects of acephate and/or methamidophos on aquatic and land animals, including humans are reviewed, as these effects promote the necessity of removing acephate from the environment. Physicochemical degradation mechanisms of acephate and/or methamidophos are explored and explained, such as photo-Fenton, ultraviolet/titanium dioxide (UV/TiO_2_) photocatalysis, and ultrasonic ozonation. Compared with physicochemical methods, the microbial degradation of acephate and methamidophos is emerging as an eco-friendly method that can be used for large-scale treatment. In recent years, microorganisms capable of degrading methamidophos or acephate have been isolated, including *Hyphomicrobium* sp., *Penicillium oxalicum*, *Luteibacter jiangsuensis*, *Pseudomonas aeruginosa*, and *Bacillus subtilis.* Enzymes related to acephate and/or methamidophos biodegradation include phosphotriesterase, paraoxonase 1, and carboxylesterase. Furthermore, several genes encoding organophosphorus degrading enzymes have been identified, such as *opd*, *mpd*, and *ophc2*. However, few reviews have focused on the biochemical pathways and molecular mechanisms of acephate and methamidophos. In this review, the mechanisms and degradation pathways of acephate and methamidophos are summarized in order to provide a new way of thinking for the study of the degradation of acephate and methamidophos.

## Introduction

Organophosphate compounds (OPs) are one of the most widely used pesticides because of their broad spectrum, specificity, and high efficiency toward insects and pests (Mulla et al., 2020). Acephate and methamidophos are two of the most common and efficient OPs that are used for pest control in agriculture (Maia et al., 2011; Kumar et al., 2015; Pan et al., 2015).

**FIGURE 1:**
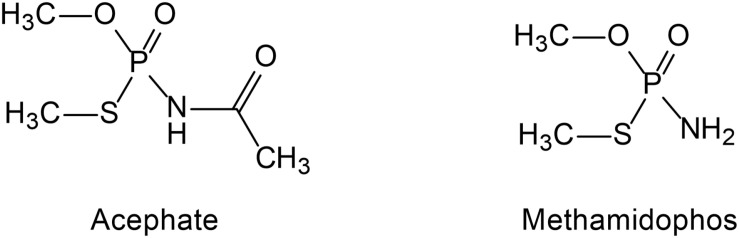
The structure of acephate and methamidophos.

Acephate [*O*, *S*-dimethyl-acetyl-phosphoramidothioate, molecular weight (MW) = 183.17] (Figure 1) is a systemic insecticide that effectively controls various pests on ornamental plants, cotton, beans, and head lettuce as well as parasites on mammalians. It is a good substitute because it is less toxic than methamidophos (Mahajna et al., 1997). Acephate is a class II “moderately hazardous” pesticide, but methamidophos is classified as a class IV “highly toxic” pesticide (World Health Organization [WHO], 2009). Acephate is highly water-soluble and can easily contaminate groundwater and soil, which is also easily absorbed by plants and accumulated in edible parts of plants (Mohapatra et al., 2011; Syed et al., 2014).

Methamidophos (*O*, *S*-dimethyl phosphoramidothioate, MW = 141.12) (Figure 1) is also a systemic insecticide effective against chewing and sucking insects and is one of the intermediate products of acephate (Farag et al., 2012; Araoud et al., 2016). Methamidophos mainly leads to enzyme inactivation by phosphorylation of the serine residues of acetylcholinesterase (AChE) and butyrylcholinesterase (BuChE) active site (Lugokenski et al., 2012). It exerts high toxicity not only through the persistent inhibition of AChE but also through the complex blocking action on neuronal nicotinic acetylcholine receptors (nAChRs), which is different from the inhibition of AChE (Di et al., 2004). In addition, methamidophos can inhibit the activity of carboxylase in cells (Mahajna et al., 1997). Because of its high efficiency, methamidophos was extensively used in large amounts in many parts of the world before 2013. Methamidophos has been restricted in China since 2013 but is still extensively used in many other developing countries (Araoud et al., 2016).

Acephate and methamidophos are used in enormous quantities because of their high efficiency in agriculture and other industrial purposes, which leads them to remain in various environments for many years (Konstantinou et al., 2006; Lugokenski et al., 2012; Dar et al., 2020). Acephate and methamidophos have both hydrophilic and hydrophobic properties that assist them easy movement between water and soil (He et al., 2017). Studies have shown that methamidophos residues may decrease the total microbial biomass carbon and fungal biomass (Li et al., 2008; Wang et al., 2008). In recent years, acephate and methamidophos have been detected in a variety of vegetables and fruits (Mohapatra et al., 2011; Syed et al., 2014; Andrade et al., 2015). At present, acephate and methamidophos have become a focus of public concern due to the harmful effects of pesticide exposure on invertebrates, mammals, and humans (Yao et al., 2018). The human body has limited metabolic capacity for acephate and methamidophos, and it may results in hyperglycemia, lipid metabolism disorder, DNA damage, increased oxidative stress, and risk of carcinogenesis after long-term exposure (Chang et al., 2009).

In recent years, many researchers have studied the different degradation processes for the removal of acephate and methamidophos from polluted environments. photo-Fenton processes, UV/TiO_2_, ultrasonic ozonation, and ionizing irradiation, etc. are widely studied for acephate or methamidophos (Dai et al., 2008; Wang et al., 2015; Yang et al., 2015; Zheng et al., 2016). Efficient and environmentally friendly microbial degradation has been considered as the most promising remediation method (Cycoń and Piotrowska-Seget, 2016; Bhatt et al., 2019; Pang et al., 2020; Feng et al., 2020; Zhang et al., 2020). Several degrading microorganisms with partial or complete degradation capacity of acephate or methamidophos have been isolated and characterized, such as *Lysinibacillus fusiformis*, *Pseudomonas* sp., *Pseudomonas pseudo alcaligenes*, *Bacillus subtilis*, *Pseudomonas azotoformans*, and *Pseudomonas putida* (Lin et al., 2016; Maddela and Venkateswarlu, 2017; Singh et al., 2017, 2020).

Furthermore, there are many studies on the degradation pathways of acephate and methamidophos as well as studies on related functional genes and enzymes (Li et al., 2007; Shen et al., 2010; Chino-Flores et al., 2012; Kumar et al., 2018). Microorganisms metabolize pesticides through their respective degradation pathways, and the metabolic capacity of microorganisms can be improved by recombination technology (Pinjari et al., 2013; Arora et al., 2017; Cycoń et al., 2017; Zhang et al., 2018; Lin et al., 2020). Microbial degradation of acephate and methamidophos is a potential tool for large-scale pollutant removal. Thus, biodegradation of acephate and methamidophos also should be concerned. We emphasize the role of physicochemical degradation and biodegradation for large-scale treatment of acephate and/or methamidophos contamination. However, few reviews have focused on the degradation mechanisms and biochemical pathways of acephate and methamidophos (Maqbool et al., 2016; Kumar et al., 2018; Jiang et al., 2019). We emphasize the role of biodegradation for large-scale treatment of acephate and/or methamidophos contamination.

This paper reviews (I) the toxicity of acephate and methamidophos, and their removal; (II) the different oxidation or reduction methods of physicochemical degradation; (III) the different types of natural degradation strains, or genetically engineered microorganisms; (IV) the organophosphorus degrading enzymes and their encoding genes.

## Toxic Effects of Acephate and Methamidophos

The extensive use and long term exposure to acephate and methamidophos results in the cumulative effect of these compounds release into the environment, which consequently results in the direct or indirect poisoning of non-target organisms. We summarize the toxic effects of acephate and methamidophos on aquatic and land animals, including humans in Table 1.

**TABLE 1:** Toxic effects of acephate and methamidophos.

No.	Pesticides	Dose and time of treatment	Study samples	Findings	References
1	Acephate	12.5–200 mg/L for 48 h	Human peripheral lymphocytes	All concentrations of acephate induced significant increase in the frequency of chromosomal aberrations (CAs) and in the formation of micronuclei (MN) dose dependently	Özkan et al., 2009
2	Methamidophos	10–100 mg/L for 96 h	Buffalo corpus luteum (CL) cells	Viable cell counts and progesterone concentration decreased significantly with dose and length of time	Afzal et al., 2011
3	Methamidophos	1–3 mg/L for 4 weeks	Mice	Methamidophos (>2 mg/L) caused the decrease of Perm motility and count in male mice and the number of live fetuses in females	Farag et al., 2012
4	Methamidophos	0.002 mg/kg for 15 days	Male mice	Methamidophos reduced the number of normal spermatozoa, weights of seminal vesicle, and testosterone levels	Maia et al., 2011
5	Acephate	0.5–4.5 mg/kg/day for 24 weeks	Rats	Acephate caused renal injury and perturbed the normal metabolic processes of rats	Hao et al., 2012
6	Acephate	5–10 mg/kg for 7 days	Earthworms	Earthworms suffered from increased lipid peroxidation, protein oxidation, DNA damage, and altered antioxidant enzyme status	Phugare et al., 2012
7	Acephate	21.3–42.6 mg/kg for 28 days	White Leghorn cockerels	Body weight ratios of immune organs were significantly suppressed	Tripathi et al., 2012
8	Methamidophos	3.75–5 mg/kg for 45 days	Mice	Methamidophos altered sperm function and DNA at different stages of spermatogenesis	Urióstegui-Acosta et al., 2014
9	Methamidophos	0.6–3 mg/L for 4 weeks	Rats	All the methamidophos-treated rats had significantly higher urea and uric acid levels	Araoud et al., 2016
10	Methamidophos	10 mg/L for 144 h	Flounders	Methamidophos evidently induced changes or damage to the flounder tissues	Peng et al., 2015
11	Methamidophos	0–80 mg/L for 72 h	Human peripheral blood mononuclear cells (PBMCs)	Methamidophos increased the generation of oxidative stress in PBMCs	Ramirez-Vargas et al., 2017
12	Acephate	2.5 mg/kg/bw in 7th–21th days	Rats	Fetal exposure to acephate may predispose offspring to type 2 diabetes and dyslipidemia during adulthood	Ribeiro et al., 2016
13	Acephate	5 μg/mL for 24 h	*Drosophila melanogaster*	Acephate caused DNA damage, cell damage, and activity change of enzymes	Rajak et al., 2017
14	Acephate	100 μg/mL for 3 h, or 200 μg/mL for 1 h	Human sperm	Acephate has cytotoxic and genotoxic effects on sperm	Dhanushka and Peiris, 2017
15	Acephate	85.2 mg/L for 4 weeks	Broiler Chicks	Acephate significantly affected blood cells and lipid profile and significantly decreased the antioxidant capacity of liver and kidneys	Farag et al., 2017
16	Methamidophos	25 and 500 μg/L for 72 h	Zebrafish	Methamidophos affects the neurodevelopmental genes and cell apoptosis in the brain	He et al., 2017
17	Acephate	0.168 mg/L or 6.97 mg/L for 48 h	Honey bees	Acephate inhibited activity of the glutathione *S*-transferase (GST), and acetylcholinesterase (AChE) at residue concentration	Yao et al., 2018
18	Acephate	0.01–100 mg/L for 48 h	Zebrafish	Acephate induced zebrafish developmental delay and malformation and decreased embryonic surface tension.	Liu et al., 2018
19	Methamidophos	5 mg/kg for 4 days	Mice	Methamidophos opens the blood–testis barrier	Ortega-Olvera et al., 2018
20	Acephate	50 mL of 50% acephate solution	Humans	Acephate and its metabolite methamidophos may cause acute lethal poisoning	Takayasu et al., 2019
21	Methamidophos	0.004 mg/kg for 15 and 50 consecutive days	Mice	Short- and long-term exposure to methamidophos impaired spermatogenesis	Carvalho et al., 2020

Zebrafish is a commonly used model test organism due to its advantages of high genetic and organ system homology to humans, external fertilization, high fecundity, and transparency in early adulthood (Liu et al., 2018). It has reported that methamidophos exposure may affect neurodevelopmental genes and activate intracellular apoptosis, leading to early developmental neurodamage in zebrafish (He et al., 2017). The brain may be an important target of methamidophos toxicity in zebrafish, revealing the potential neurotoxicity of methamidophos to other aquatic species and humans (Rodríguez et al., 2012; Peng et al., 2015; He et al., 2017). Zebrafish embryo development retardation, larval deformities, and decreased chorionic surface tension were also induced by exposure to acephate (Liu et al., 2018). Since acephate and methamidophos are prone to accumulate in water, large quantities of residual pesticides are frequently exposed to and ingested by aquatic organisms. Water is the source of life, and pesticide residues in water can also pass to other living beings through the food chain.

*Drosophila* is also a model test organism that has amazing similarities in gene structure and function with higher vertebrates. Acephate contamination at higher concentrations than 5 g/mL can cause DNA damage and mortality in the fruit fly, and it may disrupt the balance of oxidase and antioxidant enzymes, such as catalase (CAT), glutathione-*S*-transferase (GST), cytochrome P450 (CYP450), AChE, and superoxide dismutase (SOD) (Rajak et al., 2017). Chronic toxicity of acephate to honeybees is characterized by weight loss and esterase inhibition (Yao et al., 2018). Earthworms exposed to soil containing acephate also experience oxidative stress, characterized by lipid peroxidation, protein oxidation, DNA damage, and changes in antioxidant enzyme status (Phugare et al., 2012). Acephate not only significantly reduces the antioxidant capacity of bird liver and kidney but also increases lipid peroxidation, interleukin and tumor necrosis factor in both organs and also affects the immune response (Tripathi et al., 2012; Farag et al., 2017). Methamidophos had a toxic effect on the number of viable counts, morphology, and histological changes of corpus luteal cells and progesterone production in bovines (Afzal et al., 2011).

Overuse of acephate and methamidophos can lead to an increase in high blood sugar, impaired metabolism, DNA damage, reproductive barriers, and cancer in rats (Maia et al., 2011; Araoud et al., 2016; Ribeiro et al., 2016). Acephate alters glucose metabolism in pregnant and lactating rats and predisposes their offspring to type 2 diabetes in adulthood (Ribeiro et al., 2016). Exposure to acephate may lead to metabolic disorders in mice, whereas changes in some endogenous metabolites lead to kidney damage and disrupt normal metabolic processes in rats, including glucose, nucleic acid, and protein metabolism (Hao et al., 2012). Methamidophos can cause lesions in the testis and epididymis, characterized by obstruction of spermatogenesis in the testis and severe edema in the epididymis, respectively (Farag et al., 2012). Methamidophos can cause DNA damage at different stages of spermatogenesis and reduce sperm quality in mice through acrosomal response and fertilization ability assessment (Urióstegui-Acosta et al., 2014). In addition, methamidophos also decreases the expression of zonula occludens protein 2 (ZO-2) in sperm cells of spermatic tubules, induces phosphorylation of ZO-2, and occludens in the testes. It also reduces the interactions between these proteins assessed by immunoprecipitation, which leads to reproductive toxicity in male mice (Ortega-Olvera et al., 2018).

The human body can be very vulnerable when exposed to methamidophos or acephate. Acephate could be a genotoxin, which would make it a severe threat to human health. It can cause chromosomal changes and DNA damage in human lymphocytes (Özkan et al., 2009). Moreover, it is suggested that acephate also exhibits cytotoxic and genotoxic effects on human sperm by disrupting sperm motility, cell membrane integrity, and sperm volume (Dhanushka and Peiris, 2017). In addition, methamidophos increases the generation of oxidative stress in human peripheral blood mononuclear cells (Ramirez-Vargas et al., 2017). The accumulation caused by the widespread use of acephate and methamidophos has led to toxic effects among many biological systems and has caused many health problems. Growing concern about the accumulation of pesticide residues in our food, soil, and wastewater has led to a great deal of interest in their removal.

## Physicochemical Degradation Methods of Acephate and Methamidophos

Physical adsorption and chemical degradation methods were first proposed to remove acephate and methamidophos from the environment. The advantages of physical adsorption and chemical degradation methods lie in their broad spectrum of pollutants, their adaptability to the environment, and their effectiveness. Advanced oxidation processes (AOPs) are a more efficient composite technology that combines the advantages of different physicochemical degradation methods to more efficiently remove methamidophos and acephate; examples of these methods include photo-Fenton process, UV/TiO_2_, and ultrasonic ozonation (Dai et al., 2008; Wang et al., 2015; Zheng et al., 2016).

### Physicochemical Degradation of Acephate

Physicochemical methods play an important role in the degradation of acephate. In the absence of surfactants, colloidal manganese dioxide has been shown to degrade acephate by oxidation (Qamruzzaman, and Nasar, 2014). Ultrasonic degradation is a physical degradation method with simple operation and few byproducts, whose main mechanisms are mechanical bond breaking and free radical reaction. When the ultrasonic power is high enough, the liquid produces instantaneous negative pressure, local high temperature, and a high-pressure environment, which finally cause water molecules to break up and become strong oxidants, such as hydrogen peroxide radicals (•OOH) and hydroxyl radicals (•OH) (Shriwas and Gogate, 2011; Golash and Gogate, 2012). Ultrasound and ozonization have synergistic effects in complex systems. The degradation efficiency of acephate ozonization can be increased from 60.6 to 87.6% in 60 min with 160 kHz ultrasound irradiation because it can improve the oxidizability of nitrogen atoms (Wang et al., 2015). Under visible light irradiation, nanocomposite Co_3_O_4_/McM-41 can completely remove acephate in 40 min (50 mg/L) or 60 min (100 mg/L) (AbuKhadra et al., 2020). Under the same conditions of Co_3_O_4_/McM-41, ZnFe_2_O_4_–TiO_2_ can remove 89.5% of acephate within 120 min, and the optimal value of the photocatalyst and H_2_O_2_ dosage of this degradation is 2.0 g/L and 8 mmol/L, respectively (Fu et al., 2012). TiO_2_ is often used as a catalyst due to its advantages of low cost, good chemical stability, and high catalytic activity. Compared to Sr/TiO_2_–PCFM, which is not modified, the modified Sr/TiO_2_–PCFM shows preferential degradation of acephate and can degrade target pollutants more quickly and efficiently (Liu et al., 2019). Furthermore, photocatalyst Fe_3_O_4_@SiO_2_@mTiO_2_ has a larger surface area and a stronger magnetic response, which can effectively degrade acephate, omethoate, and other organophosphorus pesticides (Zheng et al., 2016). The photocatalytic degradation rate of acephate (675 g.a.i./h) was shown to be 93.5% in a field trial after adding cerium-doped nano titanium dioxide (TiO_2_/Ce) (2400 g.a.i./h) for 20 h (Liu et al., 2015). TiO_2_ is a potential and efficient chemical catalyst for the removal of environmental pesticide residues, which deserves further study. In addition, a combination of multiple degradation methods may improve the degradation efficiency.

**FIGURE 2:**
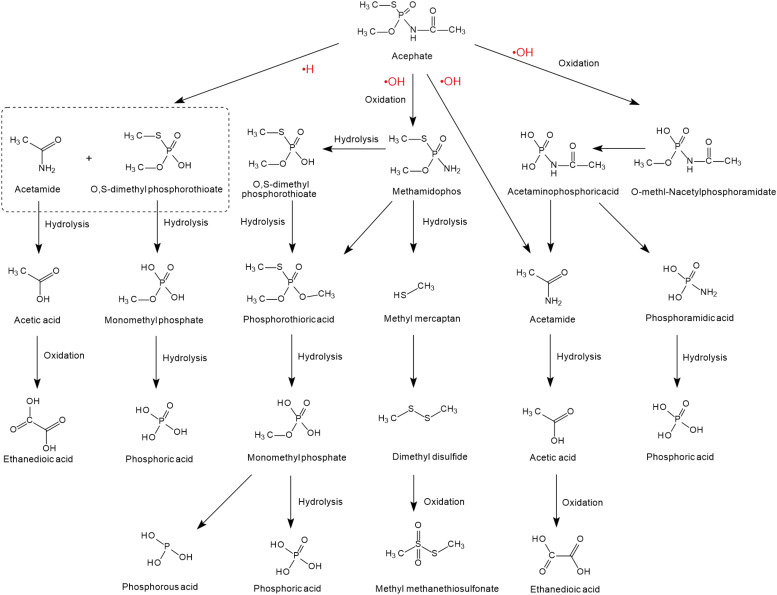
The degradation pathways of acephate reacted with •H and •OH (based on Liu et al., 2015; Wang et al., 2015; Huang et al., 2018; AbuKhadra et al., 2020).

The degradation of acephate mainly involves the destruction of N-C, P-N, P-S, or P-O bonds and the generation of the main intermediate products *O*-methl-*N*-acetylphosphoramidate and the more toxic methamidophos (as shown in Figure 2). Previous researchers have used ^60^Co irradiation, which has led to many studies on the degradation efficiency, degradation pathways, and degradation dynamics (Sun et al., 2015; Yang et al., 2015; Huang et al., 2018). Chemical degradation mainly results in the generation of free radicals in the medium, such as $e_{a\,q}^{-}$ and negative hydrogen ions (•H) and hydroxyl radicals (•OH) (Yang et al., 2015). Reducing and oxidizing free radicals play different roles in the degradation of acephate and methamidophos, so negative hydrogen ions (•H) and hydroxyl radicals (•OH) have different reaction kinetics from acephate, which leads to two different degradation pathways of acephate (Zhao et al., 2009). Negative hydrogen ions (•H) degrade acephate more quickly but produce fewer inorganic ions, while hydroxyl radicals (•OH) catalyze over more steps to produce more inorganic ions. As nucleophiles, negative hydrogen ions (•H) may first attack the P = O and C = O bonds, causing the electrons to delocalize and form temporary conjugated systems in the O-P-N-C-O bonds. When the P-N bond is disconnected, the acephate is subsequently formed with ammonium ions (${\text{NH}}_{4}^{+}$) and formic acid. At the same time, the C-S and C-O bonds of the product methamidophos are broken successively, resulting in phosphoric acid, sulfate radical, and a methyl group. In contrast, hydroxyl radicals (•OH) are strong electrophilic reagents that first attack the negatively charged S, O, and N atoms of acephate. As a result, the fracture of the P-N bond produces acetamide and phosphoric acid. The break between the P-N bond and the P-S bond produces acetaminophosphoric acid, which then produces acetamide, phosphoric acid, or ethanedioic acid. Whether in the presence of negative hydrogen ions (•H) or hydroxyl radicals (•OH), acephate will eventually be completely degraded to inorganic salts.

### Physicochemical Degradation of Methamidophos

Many physicochemical degradation methods have been developed for the degradation of methamidophos, which is very important for the removal of pesticide residues in the environment. Reductive hydrogen ions radicals (•H) can catalyze the degradation of acephate, but they cannot catalyze the degradation of methamidophos (Zhao et al., 2009). In contrast, oxidizing hydroxyl radicals (•OH) can catalyze not only the degradation of acephate but also the degradation of methamidophos (Zhao et al., 2009). Gamma irradiation can effectively degrade methamidophos into inorganic ions in aqueous solution by promoting the generation of hydroxyl radicals (•OH) in water (Zhao et al., 2009). Combined use of multiple degradation methods is a trend to improve degradation efficiency. The catalysis of methamidophos by UV and TiO_2_ has been studied. Under UV irradiation, nano TiO_2_ catalyzed 95% degradation of methamidophos within 4 h (Dai et al., 2008). In another study, the best degradation rate of methamidophos in water using nano TiO_2_ in the first 5 min reached 83.55%, which was significantly faster than the degradation reported by other studies (Zhang et al., 2006). It was found that the degradation efficiency increased with the increasing temperature and pH of the medium when the optimal amount of TiO_2_ was 12 g/L (Liu et al., 2009). It was also proved that the microwave degradation method is an effective method to remove methamidophos. In the presence of K_2_S_2_O_8_, over 96% of methamidophos can be degraded by microwave irradiation within 6 min, while the MW/K_2_S_2_O_8_ corresponding kinetic equations, half-life (*t*_1/2_) and the pseudofirst-order rate constants are lnC = −0.757t + 5.9, R^2^ = 0.9776; 0.916 min and 0.757/min, respectively (Zhang et al., 2009). Even in acidic environments, the electrolysis of the Pb/PbO_2_ electrode can better facilitate the removal of methamidophos (Martiìnez-Huitle et al., 2008). Electrode materials play a key role in electrolysis and influence the type of byproducts; thus, subsequent research on electrode materials is of great significance for electrochemical degradation. In addition, an adsorption material with good removal performance of methamidophos was obtained through modifying the structure and morphology of natural zeolite with 25 mmol/L hexadecyl-trimethyl-ammonium bromide (HDTMA) (Alvarez-García et al., 2020). Adsorption is considered to be a low-cost and simple water separation process, but it still needs to improve its material structure to enhance its adsorption capacity.

**FIGURE 3:**
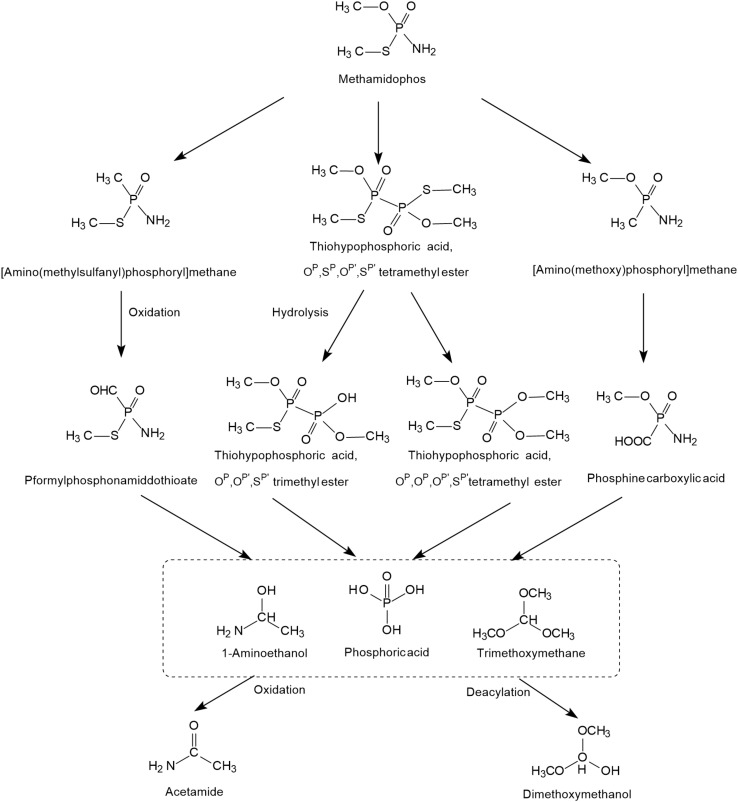
The photodegradation pathways of methamidophos catalyzed by TiO_2_ (based on Dai et al., 2008; Martiìnez-Huitle et al., 2008).

The possible photodegradation pathways of methamidophos catalyzed by TiO_2_ is shown in Figure 3 and first involves the cracking of P-S, P-N, and P-O bonds. When the P-S and P-O bonds of methamidophos are broken, P atoms react with methyl radicals to form [amino (methylsulfanyl) phosphoryl] methane and [amino (methoxy) phosphoryl] methane, which are further oxidized to *P*-formylphosphonamiddothioate and phosphine carboxylic acid, respectively. When the P-N bond of methamidophos is broken, an *O*, *S*-dimethyl phosphonothioate dimer is generated, which is more prone to further degradation. The intermediate products 1-aminoethanol and trimethoxymethane form the products acetamide and dimethoxymethanol, respectively, resulting in complete mineralization. Phosphoric acid is the main final product.

## Potential Microorganisms in Acephate and Methamidophos Degradation

There is growing interest in microbiological degradation technologies because of the risk to life of pesticide residues, which provide cheap and efficient acephate and methamidophos detoxification to complement expensive chemical methods. Microbial degradation is a potential way to decontaminate pesticide-contaminated sites (Chen et al., 2015; Yang et al., 2018; Zhan et al., 2018; Huang et al., 2019; Bhatt et al., 2020a). Biodegradation microorganisms including bacteria, fungi, actinomycetes, yeasts, and algae can be obtained by enrichment culture and recombination technology (Pinjari et al., 2013; Chen et al., 2014; Birolli et al., 2019; Bhatt et al., 2020b). At present, many researchers are looking for effective acephate or methamidophos degrading microorganisms through enrichment culture, including sewage treatment systems, organophosphorus contaminated areas, industries, and agricultural fields (Chen et al., 2012; Gao et al., 2012; Li et al., 2014; Mohan and Naveena, 2015; Lin et al., 2016). To date, only a small number of microorganisms have been isolated and identified that can fully mineralize or degrade acephate or methamidophos, as shown in Table 2.

**TABLE 2:** Microbial degradation of acephate and methamidophos.

No.	Strains or community	Sample sources	Detected metabolites	Findings	References
1	*Hyphomicrobium* sp. MAP-1	Methamidophos-contaminated soil	*O*, *S*-dimethyl phosphorothioate; *O*-methyl phosphoramidate; *S*-methyl phosphoramidate	Methamidophos (3000 mg/L) can be used as the sole carbon, nitrogen, and phosphorus source for growth and can be completely degraded in 84 h	Wang et al., 2010
2	*Penicillium oxalicum* ZHJ6	Methamidophos-contaminated soil	*O*, *S*-dimethyl phosphorothioate; *O*-methyl phosphoramidate	99.9% of methamidophos (1000 mg/L) was degraded in 12 days	Zhao et al., 2010
3	*Luteibacter jiangsuensis* sp. JW-64-1T	Methamidophos-manufacturing factory	/	60% of methamidophos (1000 mg/L) was degraded in 96 h	Wang et al., 2011
4	*Pseudomonas* sp. Ind01	Activated sludge	Methamidophos; *O*, *S*-dimethyl phosphorothioate; *O*, *O*-dimethyl phosphoramidate; *O*-methyl phosphoramidate	The bacterium can fully utilize 10 mM acephate within 15 h, and can tolerate 80 mM acephate	Pinjari et al., 2012
5	Consortium of *Exiguobacterium* sp. BCH4 and *Rhodococcus* sp. BCH2	Agricultural soil	Methamidophos; *O*, *S*-dimethyl hydrogen thiophosphate; *S*-methyl dihydrogen thiophosphate; methyl dihydrogen phosphate	75.85% of acephate (50 mg/L) was degraded within 6 days	Phugare et al., 2012
6	*Pseudomonas aeruginosa* Is-6	Agricultural soil	Methamidophos; *O*, *S*-dimethyl phosphorothioate	50 mg/L of methamidophos was completely degraded within 72 or 96 h, respectively	Ramu and Seetharaman, 2014
7	*Pseudomonas azotoformans* ACP1; *Pseudomonas aeruginosa* ACP2; *Pseudomonas putida* ACP3	Industrial soil, India	Methamidophos; *S*-methyl *O*-hydrogen phosphorothioamidate; phosphenothioic *S*-acid; phosphenamide	The strain can degrade acephate effectively under the influence of metal ions and humic acid	Singh et al., 2020
8	*Pseudomonas* sp.	Soil	/	This strain can degrade acephate and buprofezin at low concentrations	Maddela and Venkateswarlu, 2017
9	*Acinetobacte*r sp., *Pseudomonas stutzeri; Pseudomonas aeruginosa*	Organophosphorus-contaminated soil	/	80% of methamidophos (500 mg/L) can be degraded in 3 days	Li et al., 2014
10	*Lysinibacillus fusiformis*, *Pseudomonas pseudoalcaligenes*, *Pseudomonas* sp., *Pseudomonas pseudoalcaligenes*, *Bacillus cereus*	Agricultural soil	/	All the bacterium can use acephate (500 mg/L) as the sole carbon source	Mohan and Naveena, 2015
11	*Bacillus subtilis* FZUL-33	Sediment of a lake	/	The bacterium can degrade acephate–Pb(II) compound contaminants	Lin et al., 2016
12	*Pseudomonas pseudoalcaligenes*PS-5	Heavy metal polluted site	/	More than 95% of acephate (100 mg/L) was degraded within 14 days	Singh et al., 2017
13	*Agrobacterium* sp. Yw12	Activated sludge	/	43.21% of methamidophos (50 mg/L) was degraded within 96 h	Wang et al., 2012
14	*Staphylococcus rosenbach*	Methamidophos-contaminated soil	/	39.8% of methamidophos was degraded in 72 h, with 0.31% of glucose and 0.09% of peptone	Zhang et al., 2013
15	*Mucor* sp.	/	/	The immobilized fungus removes high concentrations of acephate	Zang et al., 2013
16	*Aspergillus* sp.	Rotten pap	/	Organophosphonates are used as the sole phosphorus source for microbial growth	Adelowo et al., 2015
17	*Enterobacter ludwigii* M2	Suburb soil, China	/	Strains can degrade organophosphorus pesticide residues	Zhao et al., 2014
18	*Brevundimonas faecalis* MA-B12, *Alcaligenes faecalis* sub sp., *parafaecalis* MA-B13, *Citrobacter freundii* TF-B21; *Ochrobactrum intermedium* TF-B23; *Ochrobactrum intermedium* DVB31; *Bacillus cereus*	Farmland soils, China	/	Biodegradation rates of organophosphorus pesticide (including methamidophos) range from 58.08 to 96.42% in 8 days	Jiang et al., 2019
19	*Sphingobium* sp.	Agricultural soil, Korea	/	Strains can degrade organophosphorus with P-S bonds	Ahn et al., 2018

### Acephate-Degrading Microorganisms

Acephate is degraded in two main ways, including through methamidophos or *O*-methyl-*N*-acetylphosphoramidate. *Pseudomonas* sp. Ind01 was able to degrade acephate by promoting the first step of acephate mineralization but could not utilize the methamidophos, which is involved in the process of *O*-methyl-*N*-acetylphosphoramidate (Pinjari et al., 2012). *Pseudomonas pseudoalcaligenes* PS-5 also completely transforms acephate into *O*-methyl-*N*-acetylphosphoramidate, even in the presence of heavy metal ions Cu^2+^, Fe^3+^, and humic acid (Singh et al., 2017). *Pseudomonas aeruginosa* Is-6, a biodegradable strain isolated from soil, can use acephate as a sole source of carbon, phosphorus, and energy and showed 92% degradation of acephate (1000 mg/L) in 7 days. This strain not only degrades acephate but is also able to efficiently degrade other pesticides like dimethoate, parathion, methyl parathion, chlorpyrifos, and malathion. Thus, *P. aeruginosa* strain Is-6 shows high degradation ability in contaminated soil, indicating its potential in environmental remediation (Ramu and Seetharaman, 2014). Mohan and Naveena (2015) isolated five bacterial strains, which are identified as *Bacillus cereus* ADI-10, *Nibacillus fusiformis* ADI-01, *Pseudomonas pseudoalcaligenes* ADI-03, *Pseudomonas* sp. ADI-04, and *Pseudomonas pseudoalcaligenes* ADI-06. These strains could efficiently grow and degrade acephate at 500 mg/L without any additional carbon source and were further utilized to analyze the mechanism of acephate degradation. In comparison to single bacterial cultures, the use of a combined culture of multiple strains in bioremediation was found to be more effective in terms of better metabolic and pollutant removal capabilities (Bhatt et al., 2020c; Li et al., 2020; Zhan et al., 2020). Phugare et al. (2012) reported the degradation of acephate by a bacterial consortium composed of *Exiguobacterium* sp. BCH4 and *Rhodococcus* sp. BCH2, which was capable of degrading 75.85% of 50 mg/L acephate. Microbial fixation is beneficial to the improvement of microbial utilization and degradation efficiency (Zang et al., 2013). In another study, recombinant *Escherichia coli* containing organophosphorus hydrolase (OPH) encoding plasmid was immobilized at a low temperature in polyvinyl alcohol (PVA) cryogel to form a biocatalyst with high activity, stability, and mechanical strength (Hong and Raushel, 1999). Fungi can be potential degrading microorganisms due to their extensive mycelium networks and low specificity of degrading enzymes, but only *Mucor* sp. has been found to be able to degrade methamidophos and acephate (Zang et al., 2013; Maqbool et al., 2016). However, many degrading microorganisms have not yet been tested in the field, which is much needed for social progress.

**FIGURE 4:**
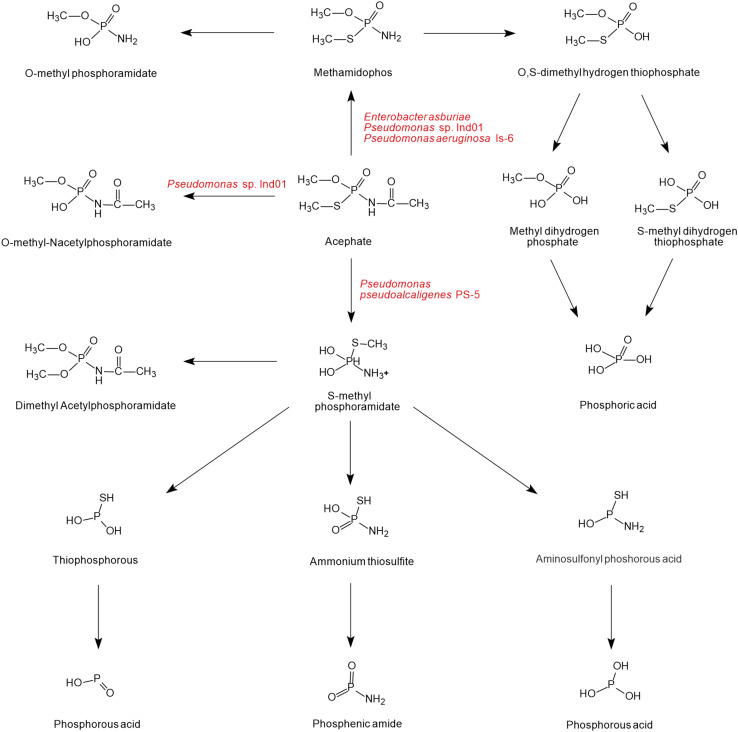
The microbial degradation pathways of acephate (based on Phugare et al., 2012; Pinjari et al., 2012; Ramu and Seetharaman, 2014; Singh et al., 2017).

As shown in Figure 4, there is a diversity of acephate degradation pathways in microorganisms. Under the catalysis of carboxylesterase enzymes, acephate first releases the acetate residue to form the main product methamidophos (Pinjari et al., 2012). The product methamidophos is then hydrolyzed to produce methyl dihydrogen phosphate, *S*-methyl dihydrogen thiophosphate, or *O*-methylphosphoramidate with the catalysis of phosphotriesterase (PTE), and eventually phosphoric acid is generated. In the process of biodegradation, PTE plays a primary role in catalyzing the first step of acephate degradation. The breaking of the P-S and P-O bonds of acephate and its intermediates is mainly dependent on the hydrolysis of PTE (Chae et al., 1994; Lai et al., 1995). With the catalysis of PTE, acephate is hydrolyzed to *O*-methyl-*N*-acetylphosphoramidate or *S*-methyl phosphoramidate. The amino group is hydrolyzed by the phosphoamide hydrolase enzyme and releases ${\text{NH}}_{4}^{+}$ ions (Ramu and Seetharaman, 2014). *S*-methyl phosphoramidate is eventually catalyzed to produce phosphorous acid, phosphenic amides, or phosphorous acid of lower molecular mass.

### Methamidophos-Degrading Microorganisms

*Hyphomicrobium* sp. MAP-1 can be grown using methamidophos as its sole source of carbon, nitrogen, and phosphorus, which can completely degrade 3000 mg/L of methamidophos within 84 h under optimum conditions (pH 7.0 and temperature 30°C) (Wang et al., 2010). *Penicillium oxalicum* ZHJ6 can degrade 99.9% of 1000 mg/L methamidophos within 12 days by co-metabolizing ethanol, glucose, fructose, sucrose, lactose, starch, and dextrin as carbon sources (Zhao et al., 2010). *Acinetobacter* sp., *Pseudomonas stutzeri*, and *Pseudomonas aeruginosa* can degrade 80% of 500 mg/L of methamidophos in 3 days under optimal conditions (pH 7.0 and temperature 30–35°C), which can not only use glucose, fructose, ethanol, and gal as carbon sources and energy but can also degrade various organophosphorus (Li et al., 2014). Isolated *Luteibacter jiangsuensis* sp. nov. can effectively use methamidophos as the sole carbon source (Wang et al., 2011). The intracellular degrading enzymes of methamidophos have been preliminarily discovered, and the optimum conditions have been explored using the response surface method (Wang et al., 2012; Zhang et al., 2013). Interestingly, some bacteria can degrade a series of organophosphorus pesticides including methamidophos, such as *Aspergillus* sp*., Enterobacter ludwigi* M2, and *Brevundimonas faecalis* MA-B12 (Zhao et al., 2014; Adelowo et al., 2015; Jiang et al., 2019). In particular, *Sphingobium* sp. K22212 and *Sphingobium* sp. Cam5-1 could only degrade organophosphorus insecticides with P-S bonds (Ahn et al., 2018). Few microorganisms can degrade acephate or methamidophos, or even completely mineralize them. Some bacteria only have partial degradation abilities, in which case, the combination of multiple strains can have a better degradation effect. Therefore, more strains capable of adapting to the environment and of high degradation need to be discovered.

**FIGURE 5:**
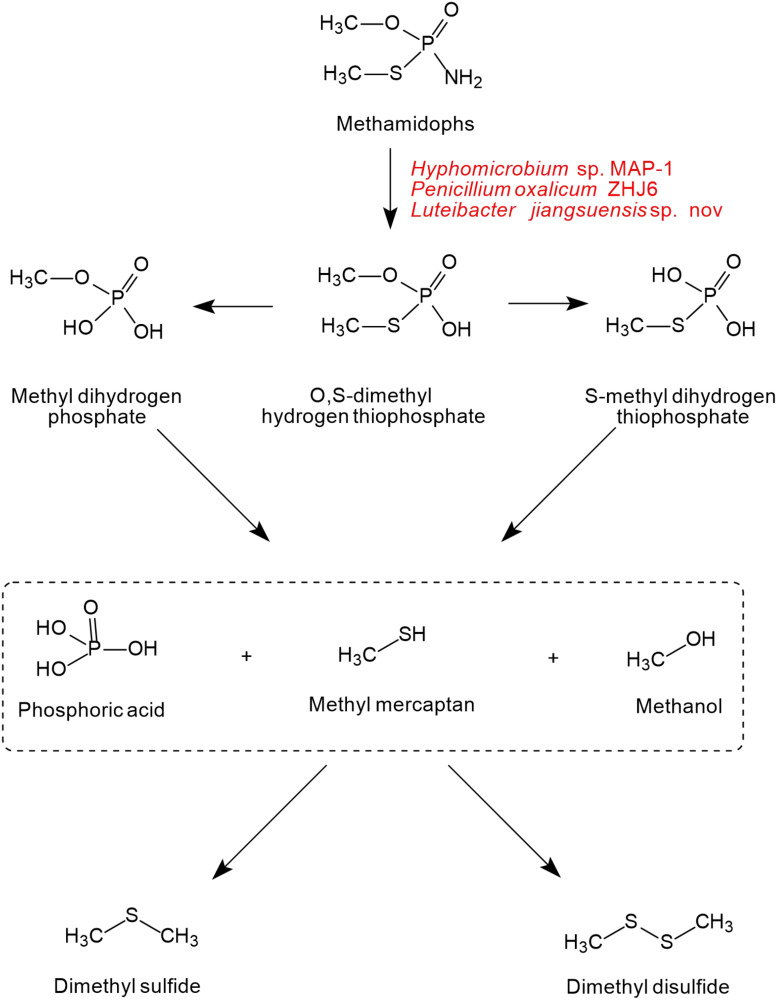
The microbial degradation pathway of methamidophos (based on Wang et al., 2010).

As shown in Figure 5, only one pathway of methamidophos degradation has been identified in microorganisms. Like acephate degradation, PTE and phosphoamide hydrolase are involved in the intracellular degradation. The P-N bond of methamidophos is first hydrolyzed by phosphoamide hydrolase. With the hydrolysis of PTE, the products *O*, *S*-dimethyl hydrogen thiophosphate generate phosphoric acid, methyl mercaptan, and methanol with the breaking of P-O and P-S bonds. In particular, methyl mercaptan in methamidophos degradation bacteria generates further dimethyl sulfide and dimethyl disulfide, which are not found in the acephate biodegradation pathway.

## Molecular Mechanism of Acephate and Methamidophos Degradation

### Degrading Enzymes of Acephate and Methamidophos

Acephate and methamidophos are highly toxic organophosphate pesticides for long term use, and their microbial degradation (including molecular and genetic mechanism) has attracted extensive attention due to its ecofriendly effectiveness (Scott et al., 2008). In addition, many biodegradable bacteria are effective in laboratory liquid media, but have poor degradation ability in field soil (Chen et al., 2013; Huang et al., 2020; Mishra et al., 2020). Therefore, more powerful enzymatic and genetic engineering techniques need to be applied. In the past, many researchers have focused on extracting PTE from microbial cells, most of which come from microorganisms and a few from other animals (Liu et al., 2017).

Microorganisms play an important role in promoting the degradation of these organophosphorus pesticides, and PTE (EC 3.1.8.1) is an effective tool to degrade organophosphorus pesticides. PTE has the stereoselectivity to degrade organophosphorus pesticides and tends to degrade (S_*p*_)-isomers of acephate and methamidophos (Chae et al., 1994; Hong and Raushel, 1999). Most studies of bacterial PTE have shown extensive pH activity, and PTE purified from two bacterial strains *Pseudomonas aeruginosa* F10B and *Clavibacter michiganensis* subsp. *insidiosum* SBL11 reached peak activity at the pH value of 9.0 (Das and Singh, 2006). Another form of PTE was isolated from *Pseudomonas monteilii*, and phosphotriestercoroxon was the only phosphorus source (Horne et al., 2002). PTE isolated from the soil bacterium *Pseudomonas diminuta* significantly promoted the hydrolysis of organophosphate triester bonds (Ghanem and Raushel, 2005). Phosphotriesterase OPHC2, isolated from *Pseudomonas pseudoalcaligenes* and belonging to the metallo-β-lactamase superfamily, had unusual heat resistance and some OP degradation ability (Gotthard et al., 2013).

**FIGURE 6:**
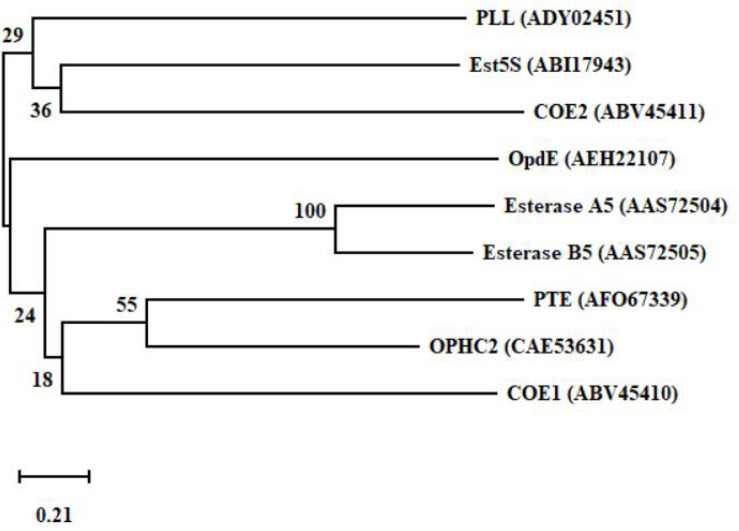
A phylogenetic tree of the key functional enzymes involved in the degradation of organophosphate pesticides. PTE was isolated from *Pseudomonas monteilii* (Horne et al., 2002). OPHC2 was isolated from *Pseudomonas pseudoalcaligenes* (Gotthard et al., 2013). PLL was isolated from *Vulcanisaeta moutnovskia* (Kallnik et al., 2014). Est5S was isolated from cow rumen bacteria (Kambiranda et al., 2009). OpdE was isolated from *Enterobacter* sp. (Chino-Flores et al., 2012). COE1 was isolated from *Bemisia tabaci* (Alon et al., 2008). COE2 was isolated from *Bemisia tabaci* (Alon et al., 2008). Esterase A5 was isolated from *Culex pipiens* (Buss and Callaghan, 2004). Esterase B5 was isolated from *Culex pipiens* (Buss and Callaghan, 2004).

The production of degrading enzymes is responsible for the resistance in many animals, and the enzymes or genes involved in organophosphorus degradation and resistance are summarized in Table 3. The evolutionary relationships among the functional enzymes involved in the degradation of organophosphate pesticides are shown in Figure 6. In addition to phosphotriesterase, other enzymes that detoxify organophosphorus pesticides are also being isolated, such as paraoxonase 1 (PON1), parathion hydrolase, and somanase (Kumar et al., 2018). As an A-esterase, PON1 can detoxify organophosphate pesticides in the phase-I metabolism of the liver. The PON1-L55M and PON1-Q192R genotypes, the two most important coding region polymorphisms of PON1, are related to the occurrence of Parkinson’s disease, Alzheimer’s disease, and amyotrophic lateral sclerosis (Androutsopoulos et al., 2011). Through quantitative polymerase chain reaction (qPCR) analysis, acetylcholinesterase 2 (AChE2) and esterase 1 (EST1) were found to be metabolic enzymes involved in the process of allogenic biological detoxification of organophosphate pesticides; increased synthesis of these enzymes can distinguish between organophosphorus-resistant and -susceptible populations (Brito et al., 2017). Acetylcholinesterase (AChE, 3.1.1.7), isolated from *Schizaphis graminum* (Rondani), is tolerant to organophosphate pesticides, and it can significantly improve the resistance ability of *Schizaphis graminum* (Gao and Zhu, 2002). A high biotechnology potential phosphotriesterase-like lactonase (PLL) has been isolated, encoded by Vmut 2255 in the hyperthermoacidophilic crenarchaeon *Vulcanisaeta moutnovskia* (VmutPLL) (Kallnik et al., 2014). A *Phe362Tyr* mutation in the AChE gene of *Lepeophtheirus salmonis* protects from pesticide toxicity (Helgesen et al., 2019). Cattle ticks, also known as *Boophilus microplus*, generally have resistance to the main organophosphate pesticides. Research that isolated acetyl cholinesterase cDNA from *Boophilus microplus* indicates that it can encode a 62 kDa protein. After translation and modification, acetylcholinesterase can develop resistance to organophosphate pesticides (Baxter and Barker, 1998). Rumen ecosystems are made up of a variety of microorganisms and protozoa that produce enzymes that help to detoxify toxic compounds in the rumen, such as organophosphate pesticides. The cow rumen bacterial esterase gene (*est5S*) is 1098 bp long, encodes a protein with 366 amino acid residues, and has a molecular weight of 40 kDa. The enzyme has great potential in cleaning up contaminated pesticides (Kambiranda et al., 2009). Enzymes isolated from insects can also be used for the biodegradation of organophosphate pesticides.

**TABLE 3:** Genes/enzymes involved in the degradation of acephate and methamidophos.

No.	Genes/enzymes	Sources	Findings	References
1	*opdE*	*Enterobacter* sp. cons002	It consists of 753 bp and encodes a protein of 25 kDa	Chino-Flores et al., 2012
2	*mpd*	*Sphingomonas* sp. Dsp-2	It is chromosome-based	Li et al., 2007
3	*ophc2*	*Pseudomonas pseudoalcaligenes* C2-1	Its size is 975 bp	Wu et al., 2004
4	*Phe362Tyr*	*Lepeophtheirus salmonis*	It is an acetylcholinesterase gene	Helgesen et al., 2019
5	*est5S*	The cow rumen-bacteria	It is an esterase gene with 1098 bp in length and a molecular weight of 40 kDa, encoding a protein of 366 amino acid residues	Kambiranda et al., 2009
6	*A5-B5*	*Culex pipiens*	It is a carboxylesterase gene; The gene spacer is 3.7 Kb	Buss and Callaghan, 2004
7	*E3*	New World screwworm	It is a carboxylesterase gene with the mutation of G137D	Da Silva et al., 2011
8	*ace1*	B-biotype of *Bemisia tabaci*	It is an acetylcholinesterase gene with one mutation of *Phe392Trp*	Alon et al., 2008
9	*ace2*	B-biotype of *Bemisia tabaci*	It is an acetylcholinesterase gene with one silent nucleotide polymorphisms	Alon et al., 2008
10	*coe1*	B-biotype of *Bemisia tabaci*	It is a carboxylesterase gene, which Overexpresses 4-fold	Alon et al., 2008
11	*coe2*	B-biotype of *Bemisia tabaci*	It is a carboxylesterase gene	Alon et al., 2008
12	Acetylcholinesterase mRNA	*Schizaphis graminum*	It is an acetylcholinesterase gene which shows 1.1- to 3.8-fold less sensitivity to inhibition by six OP compounds	Gao and Zhu, 2002
13	Acetylcholinesterase cDNA	*Boophilus microplus*	It encodes 62 kDa protein which related to organophosphorus pesticide resistance	Baxter and Barker, 1998
14	Acetylcholinesterase	*Plutella xylostella*	It is an acetylcholinesterase with the gene of A298S and G324A mutations	Sonoda and Igaki, 2010
15	Phosphotriesterase	*Pseudomonas diminuta* and *Flavobacterium* sp.	It is a monomeric metallo-enzyme with a molecular weight of 36,000, which catalyzes the cleavage of the phosphorus-oxygen bond in organophosphate triesters	Chae et al., 1994
16	OPHC2	*Pseudomonas pseudoalcaligenes*	It is a phosphotriesterase, belonging to the metallo-β-lactamase superfamily	Gotthard et al., 2013
17	Phosphotriesterase-like lactonase	*Vulcanisaeta moutnovskia*	It is characterized as a 82 kDa homodimer and converts lactones and acyl-homoserine lactones (AHLs) with comparable activities	Kallnik et al., 2014
18	Paraoxonase 1	Animal	It is an A-esterase and Phase-I enzyme, which is involved in the hydrolysis of organophosphate esters	Androutsopoulos et al., 2011
19	Acetylcholinesterase 2	*Rhipicephalus microplus*	Distinguish between organophosphorus resistant and susceptible populations	Brito et al., 2017
20	Esterase 1	*Rhipicephalus microplus*	Distinguish between organophosphorus resistant and susceptible populations	Brito et al., 2017

Enzymes that are fixed can generally be used to improve efficiency. In the presence of appropriate immobilization, the degradation effect of the degrading enzyme is slightly better than that of the free enzyme, with a higher reuse rate and easier preservation (Jesionowski et al., 2014; Doraiswamy et al., 2019). After a series of tests, it was determined that Wax Encapsulated Organophosphate Hydrolase (WEOH) can be retained for 1 year under normal temperature, with an activity reduction of 10% (Muthyala and Gundala, 2019). More methods to improve the degradation efficiency and reuse efficiency of enzymes need to be developed.

### Genes Encoding Degrading Enzymes of Acephate and Methamidophos

Biodegradability is determined by genes, and genetic engineering technology may be used to carry out gene recombination and to enhance biodegradability. Therefore, it is very important to extract resistance genes. To date, many genes encoding organophosphorus degrading enzymes have been found, which will be of great support for the promotion of organophosphorus biodegradation, such as *opd*, *mpd*, and *ophc2*.

The gene *opd* is a widely studied gene. The *opdE* gene has been isolated from *Enterobacter* sp. cons002, which has a wide range of degrading activities against organophosphorus pesticides (Chino-Flores et al., 2012). The gene *opdE* consists of 753 bp and encodes a protein of 25 kDa (Chino-Flores et al., 2012). The sequential mutation and screening of OPH encoding genes in specific codons by saturation mutagenesis can improve the degradation ability of OPH mutated enzymes on the P-S bonds of organophosphorus pesticides (Schofield and DiNovo, 2010). Through genetic engineering technology, the *opd* gene cloned from *Brevundimonas mututa* was isogenously expressed in the acephate-mineralizing strain *Pseudomonas* sp. Ind01, suggesting that the engineered strain could degrade a variety of OP insecticides (Pinjari et al., 2013).

The *mpd* and *ophc2* genes are also very important in degradation. The gene *mpd* has a low similarity with the *opd* gene, and the *mpd* gene encoded enzyme can degrade organophosphorus pesticides such as parathion and fenitrothion (Cui et al., 2001). The *mpd* gene was first cloned from *Plesiomonas* sp. strain M6 and then cloned from *Sphingomonas* sp. strain dsp-2 (Cui et al., 2001; Li et al., 2007). The gene *ophc2* has a size of 975 bp, and its homology is less than 50% compared with other organophosphorus hydrolase genes in GenBank (Wu et al., 2004). The gene *ophc2* was successfully heterogeneously expressed in *Pichia pastoris* and *Escherichia coli* by gene cloning (Chu et al., 2006; Shen et al., 2010). The emergence of metagenomics technology will rapidly promote the discovery of degrading microorganisms at the gene level and gradually replace the arduous enrichment and culture method (Jeffries et al., 2018). With the increase of people’s knowledge of the application of enzymes, more and more new biocatalysts with functional properties have been studied.

### Acephate and Methamidophos Resistance

Due to the frequent use of acephate/methamidophos in agriculture over a long period of time, insect resistance to insecticides is on the rise. There are two molecular mechanisms for *Plutella xylostella* resistance, including the reduction in target sensitivity and the increase in detoxification. The acephate resistance of resistant varieties of diamondback moth *Plutella xylostella* was 47 times higher than that of sensitive varieties because of the metabolic detoxification mediated by GST and sensitivity reduction of AChEs (Sonoda and Igaki, 2010). Further research found that A298S and G324A mutations in AChE1 of *Plutella xylostella* also reduced the sensitivity to methamidophos (Sonoda and Igaki, 2010). In addition, CYP450 can directly detoxify OP compounds by catalyzing oxidation/reduction reactions, and esterase can hydrolyze OP pesticides. In a laboratory study, the increase of esterase activity was an important reason for acephate resistance enhancement in Indian brown planthoppers (Malathi et al., 2017).

There is a significant correlation between mutation frequency and resistance level in natural populations. The first two carboxylesterase genes (*coe1* and *coe2*) were isolated from *Bemisia tabaci*, in which *coe1* was overexpressed (about 4-fold) in the organophosphate-resistant strain. Sufficient data and biochemical evidence support that increased *coe1* and *coe2* activity is involved in *Bemisia tabaci* resistance to organophosphate insecticides (Alon et al., 2008). The resistance of organophosphate in biotype B is closely related to the point mutation in *ace1* and *ace2* acetylcholinesterase. By comparison of nucleic acids and derived amino acid sequences, there are only silent nucleotide polymorphisms in the *ace2* strain and only one mutation in the *ace1* strain, namely, *Phe392Trp* (Phe331 in *Torpedo californica*) (Alon et al., 2008). Under pressure from organophosphate pesticides, G137D mutations in the carboxylesterase E3 gene in the New World screwworm are associated with a general form of resistance to acetylcholinesterase through the metabolic detoxification of pesticides (Da Silva et al., 2011). Carboxylesterase gene amplification can increase the organophosphate resistance of *Culex pipiens*. The amplified carboxylesterase gene of *Culex pipiens* resistance was originally collected in Cyprus. Genomic DNA fragments containing two loci encoding the carboxylesterase alleles *A5* and *B5* have been cloned and sequenced; the gene spacer is 3.7 Kb in length in the *A5-B5* amplituter (Buss and Callaghan, 2004). In the case of continuous use of organophosphate pesticides, some organisms will become more and more resistant to them.

In addition, insect resistance can also be induced under artificial conditions. One researcher screened 25 generations of mutant *Laodelphax striatellus* to obtain insect cross-resistance to acephate, deltamethrin, imidacloprid, methomyl, carbosulfan, and diazinon (Xu et al., 2014). In addition, the cross resistance of *Laodelphax striatellus* may also be related to the participation of CYP450s and esterases, and the overexpression of *CYP6AY3v2*, *CYP306A2v2*, *CYP353D1v2*, and *LSCE36* genes (Xu et al., 2014). Insecticide-detoxifying carboxylesterase (CE) was shown to metabolize acephate and is controlled by the gene *CpCE-1* of wild *Cydia pomonella* (CP) (Yang et al., 2014). Earthworms promote the detoxification of methamidophos mainly through their own biosorption and syntropy, especially the biodegradation of enzymes released in the soil (Zhou et al., 2008). The emergence of insect resistance is the result of organisms gradually adapting to acephate and methamidophos, which is beneficial to the extraction of resistance genes and the screening of new biodegradation agents.

## Conclusion and Future Perspectives

In modern agricultural production, acephate and methamidophos play important roles in controlling pests and increasing the crop yield. However, improper use of acephate and methamidophos can lead to critical environmental pollution and life-threatening health problems due to the high toxicity of methamidophos to non-target organisms. Many physicochemical methods have been developed and applied for the remediation of acephate and methamidophos, but they are thought to be too expensive to use on a large scale. Therefore, microbial degradation is considered to be a better and effective degradation method for acephate and methamidophos. Microorganisms capable of metabolizing acephate and methamidophos have been isolated, but further studies are needed, such as the continued development and utilization of degrading enzymes, the development of engineered degrading microorganisms, and overcoming environmental complexity. Microbial degradation kinetics, degradation pathways, and related enzymes and functional genes need to be further studied, as this is conducive to the *in situ* repair of methamidophos and methamidophos. Therefore, in order to understand the genetic analysis of acephate and its related catabolic genes, we need to use advanced molecular technologies such as metagenomics, proteomics, and transcriptome analysis to reveal the missing links and evolutionary mechanism and metabolic pathway involved in the process of bio-degradation. In the future, metagenomics will serve as a useful tool to predict microbial degradation in polluted habitats and may facilitate the practical application of acephate and methamidophos degrading microorganisms from various contaminated sites.

## Author Contributions

SC conceived of the presented idea. ZL contributed to the writing and prepared the figures and tables. SP, WZ, SM, PB, and SC participated in revising the manuscript. All the authors approved it for publication.

## Conflict of Interest

The authors declare that the research was conducted in the absence of any commercial or financial relationships that could be construed as a potential conflict of interest.
